# Number of natural teeth and intrinsic capacity among hospitalized older adults in China: a cross-sectional study on the role of social support

**DOI:** 10.3389/fmed.2026.1848888

**Published:** 2026-08-21

**Authors:** Xiaoming Zhang, Xinjuan Wu

**Affiliations:** 1 Department of Emergency, The People's Hospital of Bao'an, Shenzhen, China; 2 Department of Nursing, Peking Union Medical College Hospital, Chinese Academy of Medical Sciences and Peking Union Medical College, Beijing, China

**Keywords:** cross-sectional study, intrinsic capacity, older adults, social support, tooth loss

## Abstract

**Background:**

Tooth loss is common in older adults and has been associated with adverse health outcomes, but its relationship with intrinsic capacity (IC), a multidimensional indicator of healthy aging, remains insufficiently understood. Whether social support is involved in this association is also unclear.

**Methods:**

This cross-sectional study included 421 hospitalized adults aged 60 years or older who were recruited from Peking Union Medical College Hospital, Beijing, between February and May 2022. The number of natural teeth was assessed by direct visual inspection of the oral cavity by a trained nurse during routine admission assessment. IC was evaluated using six components derived from five World Health Organization domains: locomotion, cognition, vitality, psychological function, hearing, and vision. Participants with impairment in at least one component (IC score ≤ 5 of 6) were classified as having declined IC. Social support was measured using the Multidimensional Scale of Perceived Social Support. Multivariable logistic regression was used to examine the association between natural-tooth count and declined IC. Exploratory structural equation modeling (SEM) was used to assess an indirect statistical pathway involving social support.

**Results:**

Among 421 participants (mean age, 71.5 ± 6.3 years; 60.6% male), 66.98% had declined IC. Compared with participants with ≥20 natural teeth, those with < 20 natural teeth had higher odds of declined IC after adjustment for sociodemographic, clinical, and behavioral factors and Charlson Comorbidity Index (OR = 3.11, 95% CI: 1.82–5.32, *P* < 0.001). When natural-tooth count was modeled continuously, each additional tooth was associated with lower odds of declined IC (OR = 0.96, 95% CI: 0.93–0.98, *P* = 0.001). In exploratory SEM using the 0–6 IC score, a significant indirect association through social support was observed (β = 0.009, 95% CI: 0.005–0.014, *P* < 0.001), accounting descriptively for 41.8% of the total statistical association.

**Conclusions:**

Fewer natural teeth were associated with declined IC among hospitalized older adults. Social support may be involved in this association, but the cross-sectional design precludes causal interpretation. These findings support incorporating oral health assessment into geriatric care and warrant longitudinal studies to clarify temporal pathways.

## Introduction

The global population is aging rapidly, with the number of individuals aged 60 years and older projected to reach 2.1 billion by 2050. In the World Report on Aging and Health (2015), the World Health Organization (WHO) introduced intrinsic capacity (IC) as a core construct of healthy aging, defined as the composite of all physical and mental capacities of an individual. IC encompasses five key domains: locomotion, cognition, vitality, psychological wellbeing, and sensory function (1). IC is conceptually distinct from functional ability, which reflects the interaction between intrinsic capacity, environmental characteristics, and personal factors. Because lower IC has been associated with disability, hospitalization, cardiovascular events, fractures, and mortality (2–5), identifying potentially modifiable correlates of IC decline is important for geriatric assessment and healthy aging.

A wide range of factors have been associated with IC decline, including advanced age, chronic diseases, lower educational attainment, and unmarried status (6). In recent years, increasing attention has been directed toward the role of oral health. The oral cavity serves not only as the entry point of the digestive system but also plays a vital role in nutritional intake, speech, and social interaction (7). Tooth loss, a common condition among older adults, directly affects masticatory efficiency, dietary choices, and nutrient absorption (8). Data from the Fourth National Oral Health Epidemiological Survey in China indicate that the prevalence of edentulism among adults aged 65–74 years is 6.8%, with a mean of only 22.5 remaining teeth (9). Accumulating evidence suggests that poor dental status is associated with a range of adverse health outcomes, including malnutrition, sarcopenia, cognitive decline, depressive symptoms, frailty, cardiovascular disease, and diabetes (10).

Several mechanisms may underlie the association between tooth loss and declines in intrinsic capacity. First, reduced masticatory efficiency may lead to inadequate dietary intake, contributing to malnutrition and subsequent declines in muscle strength and physical function (11). Second, periodontal disease and oral inflammation may promote systemic inflammation, thereby accelerating biological aging and impairing multiple physiological systems (12). Third, tooth loss may negatively affect speech, appearance, and social participation, increasing the risk of social isolation and psychological distress, which may in turn compromise overall functional capacity (13).

To date, only a limited number of studies have examined the relationship between oral health and intrinsic capacity. Evidence from analyses of the NHANES database indicates that reduced dentition, edentulism, and periodontal disease are associated with impairments across multiple IC domains (14–16). For example, edentulous individuals have been reported to have a significantly higher likelihood of functional impairment, including an increased risk of sensory deficits such as hearing impairment (14). More recent studies have also linked self-reported oral health problems to declines in several IC domains (16). However, these studies were conducted exclusively among community-dwelling older adults in the United States and primarily focused on domain-specific outcomes rather than IC as an integrated construct.

Importantly, declines in intrinsic capacity are unlikely to result from a single factor but instead arise through complex interactions among biological, behavioral, and social processes. Social support, defined as the perceived or actual availability of emotional, informational, and instrumental resources from social networks, represents a key external determinant of health in older age (17). According to Socioemotional Selectivity Theory, older adults prioritize emotionally meaningful relationships, making social support particularly salient in later life (18). It is plausible that social support is linked to oral health-related behaviors, healthcare utilization, psychological distress, and functional reserve, although the present cross-sectional design cannot establish these pathways as causal mechanisms. Nevertheless, evidence integrating dental status, social support, and intrinsic capacity remains scarce, and the potential role of social support in this relationship has not been fully explored. Hospitalized older adults may differ from community-dwelling populations because acute illness, multimorbidity, polypharmacy, nutritional vulnerability, and reduced opportunities for social participation may amplify links among oral health, psychosocial resources, and IC. Understanding these relationships in this context may therefore provide clinically relevant information for inpatient geriatric assessment and discharge planning.

Therefore, this study aimed to (1) examine the association between the number of natural teeth and intrinsic capacity among hospitalized older adults in China and (2) explore the potential role of social support in this relationship. We hypothesized that a lower number of natural teeth would be associated with a higher likelihood of intrinsic capacity decline and that this association may be partly explained by differences in social support.

## Methods

### Study design and participants

This cross-sectional study was conducted at Peking Union Medical College Hospital (PUMCH), a tertiary academic medical center in Beijing, China. Participants were recruited from inpatient wards between February and May 2022. The inclusion criteria were: (1) age ≥60 years; (2) able to communicate and complete assessments; (3) voluntary participation with written informed consent. Exclusion criteria included: (1) severe cognitive impairment; (2) terminal illness with life expectancy < 6 months; (3) inability to complete assessments due to acute medical conditions.

Of the 450 eligible patients approached, 421 agreed to participate (response rate: 93.6%). All participants provided written informed consent prior to data collection. The study protocol was approved by the Ethics Committee of Peking Union Medical College Hospital (Approval No. S-K1872) and registered with the Chinese Clinical Trial Registry (ChiCTR2100055046).

### Sample size calculation

*A priori* power analysis was conducted using G^*^Power (version 3.1.9.7) for logistic regression. Based on previous literature suggesting an odds ratio of approximately 2.0 for the association between tooth loss and functional outcomes, with an alpha of 0.05 (two-tailed), power of 0.80, and assuming a medium effect size (Cohen's *f* ^2^ = 0.15) with up to 15 covariates, the minimum required sample size was calculated to be 385 participants. Our final sample of 421 participants exceeded this requirement, providing adequate statistical power.

### Assessment of number of natural teeth

The number of natural teeth was assessed by direct visual inspection of the oral cavity by a trained nurse during the routine admission assessment, rather than by participant self-report or medical or dental records. Third molars were excluded from the tooth count. The assessment was designed solely to determine the number of remaining natural teeth and did not constitute a comprehensive oral or dental examination. Accordingly, participants were categorized as having < 20 or ≥20 natural teeth using a commonly applied epidemiological threshold (19). This classification reflects anatomical tooth count only and was not intended to represent functional dentition, functional tooth units, occlusal support, or masticatory performance. No formal examiner calibration or inter-rater reliability assessment was performed because tooth counting was conducted as part of routine clinical care rather than a standardized research dental examination. In addition, detailed oral health information, including retained roots, non-functional teeth, dental implants, fixed prostheses, removable dentures, periodontal status, dental caries, oral pain, occluding pairs, masticatory performance, and reasons for tooth loss, was not available in the study database.

### Definition of intrinsic capacity

Intrinsic capacity (IC) was defined in accordance with the World Health Organization (WHO) framework, which conceptualizes IC as the composite of an individual's physical and mental capacities across five domains, including locomotion, cognition, vitality, psychological function, and sensory function (20). Because no universally accepted operational definition of IC is currently available, we selected indicators that were routinely collected during inpatient geriatric assessment and that have been widely used or clinically justified in geriatric research. Cognition was assessed using the Mini-Mental State Examination (MMSE), with a score ≤ 24 indicating cognitive impairment (21). Psychological function was evaluated using the 15-item Geriatric Depression Scale (GDS-15), with a score ≥5 considered indicative of impairment (22). Locomotion was defined according to the Asian Working Group for Sarcopenia (AWGS) criteria, with handgrip strength < 28 kg in men or < 18 kg in women indicating impaired locomotion (23). Vitality was defined as a body mass index (BMI) ≤ 18.5 kg/m^2^ (5). Sensory function was assessed using two separate components, self-reported hearing impairment and self-reported vision impairment, because these deficits may have distinct functional consequences.

Each unimpaired component was assigned a score of 1, and each impaired component was assigned a score of 0. Although the WHO framework comprises five domains, hearing and vision were treated as two separate sensory components in this study. Accordingly, the total IC score ranged from 0 to 6. In line with previous studies, participants with a total IC score ≤ 5 (indicating impairment in at least one component) were classified as having declined IC, whereas those with a score of 6 were considered to have normal intrinsic capacity (24). As sensitivity analyses, we additionally evaluated a more stringent definition of declined IC (score ≤ 4, indicating at least two impaired components) and modeled the IC score as a continuous outcome.

### Assessment of social support

Social support was measured using the Multidimensional Scale of Perceived Social Support (MSPSS), a widely validated 12-item instrument assessing perceived support from three sources: family, friends, and significant others. Each item is rated on a 7-point Likert scale (1 = very strongly disagree to 7 = very strongly agree), yielding a total score ranging from 12 to 84, with higher scores indicating greater perceived social support (25). The Chinese version of MSPSS has demonstrated excellent psychometric properties in older adult populations (26).

### Covariates

The following covariates were collected: age, sex, body mass index (BMI), number of medications (polypharmacy), hospital department (gastroenterology, cardiology, endocrinology, urology, vascular surgery, or geriatrics), educational level (primary school, middle school, or high school and above), marital status (married or other), living arrangement (living alone or not), smoking and drinking status (former/current/never), self-reported loneliness, and the Charlson Comorbidity Index (CCI).

### Statistical analysis

Participant characteristics were summarized as means ± standard deviations (SDs) for continuous variables and frequencies (percentages) for categorical variables, stratified by the number of natural teeth (< 20 vs. ≥20 natural teeth). Between-group differences were assessed using the independent-samples *t*-test for continuous variables and the chi-square test or Fisher's exact test for categorical variables, with Fisher's exact test used when the expected cell count was < 10.

Covariates were selected *a priori* using a conceptual causal framework rather than univariate *P*-values. The primary multivariable model adjusted for department, sex, age, education, marital status, living arrangement, smoking status, drinking status, polypharmacy (defined as the use of at least five medications), and Charlson Comorbidity Index. These variables were considered potential common causes, or proxies for common causes, of natural-tooth count and intrinsic capacity. BMI was not included because BMI was used to define the vitality component of IC. Social support was treated as a hypothesized mediator and was therefore excluded from models estimating the total association. Feelings of loneliness were excluded from the primary model because of their conceptual overlap with social support and the psychological IC domain. Three hierarchical logistic regression models were constructed to examine the association between the number of natural teeth and IC decline: Model 1 (unadjusted), Model 2 (adjusted for sociodemographic factors, including department, age, sex, education, and marital status), and Model 3 (further adjusted for clinical and behavioral factors, including living arrangement, smoking status, alcohol consumption, polypharmacy and Charlson Comorbidity Index). The number of natural teeth was analyzed both as a categorical variable (< 20 vs. ≥20 teeth) and as a continuous variable (per-tooth increase).

To explore whether social support statistically accounted for part of the association between the number of natural teeth and intrinsic capacity, we fitted an observed-variable structural equation model using the lavaan package in R. The observed IC score, ranging from 0 to 6 with higher values indicating better intrinsic capacity, was specified as a continuous outcome. The binary declined-IC variable was not used in this model.

The model simultaneously estimated: (1) the association between the number of natural teeth and social support (path a); (2) the association between social support and the IC score after adjustment for tooth count (path *b*); and (3) the direct association between the number of natural teeth and the IC score after adjustment for social support (path *c*'). The indirect association was calculated as *a* × *b*, and the total association as *c*' + *a* × *b*.

Both the mediator and outcome equations were adjusted for department, sex, age, education, marital status, living arrangement, smoking status, drinking status, polypharmacy (at least five medications), and Charlson Comorbidity Index. Categorical covariates were represented by indicator variables. Social support was specified as the mediator. BMI was excluded because it contributed to the vitality component of the IC outcome, and loneliness was excluded because of its potential overlap with social support and psychological function.

Parameters were estimated using robust maximum likelihood estimation. Percentile 95% confidence intervals were calculated using 5,000 non-parametric bootstrap samples generated by resampling participants with replacement. Because exposure, mediator, and outcome were assessed cross-sectionally, the model was interpreted as an exploratory decomposition of statistical associations and not as evidence of temporal or causal mediation.

ROC curve analysis was performed as an exploratory assessment of the cross-sectional discrimination of the number of natural teeth for declined IC, rather than as evidence of clinical screening utility. The area under the curve (AUC) was calculated. All statistical analyses were conducted using R software (version 4.5.1). A two-sided *P*-value < 0.05 was considered statistically significant.

### Sensitivity analyses

We conducted four pre-specified sensitivity analyses to evaluate the robustness of our findings. First, we applied a more stringent definition of declined intrinsic capacity (IC), defined as impairment in at least two IC components (IC score ≤ 4), and additionally analyzed the IC score as a continuous outcome.

Second, to align more closely with the original WHO IC framework, we combined hearing and vision into a single sensory domain to construct a five-domain IC score. Using this alternative IC structure, we evaluated four outcomes: (1) any impaired IC domain (IC score ≤ 4), (2) impairment in at least two IC domains (IC score ≤ 3), (3) the continuous five-domain IC score, and (4) the continuous number of impaired IC domains. For all analyses, the number of natural teeth was modeled both categorically (< 20 vs. ≥20 natural teeth) and continuously (per additional tooth).

Third, we performed domain-specific analyses by fitting separate multivariable logistic regression models for impairment in each IC domain (locomotion, cognition, vitality, psychological function, and sensory function). To account for multiple comparisons, Benjamini–Hochberg false discovery rate (FDR) correction was applied across the five domain-specific outcomes separately for each tooth-count exposure.

Fourth, because Mini Nutritional Assessment–Short Form (MNA-SF) data were available, we performed an additional sensitivity analysis using MNA-SF-defined vitality instead of the BMI-based vitality indicator. An MNA-SF score of 12–14 was classified as preserved vitality (normal nutritional status), whereas a score of 0–11 indicated impaired vitality (malnutrition or risk of malnutrition). Using this alternative vitality definition, we reconstructed both the original six-component IC score and the WHO-aligned five-domain IC score. For each scoring structure, we evaluated four outcomes: any impairment, impairment in at least two components/domains, the continuous IC score, and the continuous number of impaired components/domains.

## Results

## Participant characteristics

A total of 421 hospitalized older adults were included in the final analysis. The mean age was 71.5 ± 6.3 years (range: 60–89), and 60.6% (*n* = 255) were male. The mean number of natural teeth was 19.29 ± 9.9, with 42.5% (*n* = 179) having fewer than 20 teeth. The mean social support score was 58.52 ± 10.89. The prevalence of deficits across intrinsic capacity (IC) domains was as follows: vitality (4.99%), psychological (30.88%), vision (19.00%), hearing (22.57%), locomotion (33.97%), and cognition (5.94%). Overall, 66.98% of participants exhibited declined IC. Detailed characteristics of the study population are presented in Table 1.

**Table 1:** Characteristics of the study population.

Number of natural teeth category	Total sample	≥20	< 20	Standardized difference	*P*-value
*N*	421	242	179		
Age, mean ± SD	71.52 ± 6.30	69.62 ± 5.21	74.07 ± 6.75	0.74 (0.54, 0.94)	< 0.001
BMI, mean ± SD	24.50 ± 3.58	25.11 ± 3.39	23.66 ± 3.67	0.41 (0.21, 0.61)	< 0.001
Number of natural teeth, mean ± SD	19.29 ± 9.95	26.65 ± 3.56	9.35 ± 6.57	3.27 (2.98, 3.57)	< 0.001
Number of medications, mean ± SD	4.15 ± 2.83	3.91 ± 2.79	4.48 ± 2.87	0.20 (0.01, 0.39)	0.042
GDS-15 score, mean ± SD	3.70 ± 2.95	2.96 ± 2.38	4.70 ± 3.32	0.60 (0.40, 0.80)	< 0.001
Cognitive function score, mean ± SD	27.38 ± 2.73	28.10 ± 2.13	26.40 ± 3.14	0.64 (0.44, 0.83)	< 0.001
MNA-SF score, mean ± SD	11.22 ± 3.05	12.32 ± 2.29	9.73 ± 3.31	0.91 (0.71, 1.11)	< 0.001
Social support score, mean ± SD	58.52 ± 10.89	62.32 ± 9.11	53.37 ± 11.01	0.89 (0.68, 1.09)	< 0.001
Department, *n* (%)				0.25 (0.05, 0.44)	0.285
Gastroenterology	121 (28.74%)	66 (27.27%)	55 (30.73%)		
Cardiology	182 (43.23%)	109 (45.04%)	73 (40.78%)		
Endocrinology	37 (8.79%)	25 (10.33%)	12 (6.70%)		
Urology	26 (6.18%)	15 (6.20%)	11 (6.15%)		
Vascular surgery	32 (7.60%)	13 (5.37%)	19 (10.61%)		
Geriatrics	23 (5.46%)	14 (5.79%)	9 (5.03%)		
Sex, *n* (%)				0.13 (−0.06, 0.32)	0.184
Male	255 (60.57%)	140 (57.85%)	115 (64.25%)		
Female	166 (39.43%)	102 (42.15%)	64 (35.75%)		
Education, *n* (%)				0.31 (0.11, 0.50)	0.009
Primary school or lower	89 (21.14%)	40 (16.53%)	49 (27.37%)		
Middle school	208 (49.41%)	120 (49.59%)	88 (49.16%)		
High school or higher	124 (29.45%)	82 (33.88%)	42 (23.46%)		
Marital status, *n* (%)				0.21 (0.02, 0.41)	0.092
Married	372 (88.36%)	220 (90.91%)	152 (84.92%)		
Never married	12 (2.85%)	7 (2.89%)	5 (2.79%)		
Other (widowed/divorced/separated)	37 (8.79%)	15 (6.20%)	22 (12.29%)		
Living arrangement, *n* (%)				0.13 (−0.06, 0.32)	0.179
With others	381 (90.50%)	223 (92.15%)	158 (88.27%)		
Alone	40 (9.50%)	19 (7.85%)	21 (11.73%)		
Smoking status, *n* (%)				0.31 (0.12, 0.51)	0.006
Never smoked	234 (55.58%)	148 (61.16%)	86 (48.04%)		
Former smoker	69 (16.39%)	29 (11.98%)	40 (22.35%)		
Current smoker	118 (28.03%)	65 (26.86%)	53 (29.61%)		
Drinking status, *n* (%)				0.07 (−0.12, 0.26)	0.768
Never drinks	266 (63.18%)	155 (64.05%)	111 (62.01%)		
Occasional drinker	61 (14.49%)	36 (14.88%)	25 (13.97%)		
Regular drinker	94 (22.33%)	51 (21.07%)	43 (24.02%)		
Feelings of loneliness, *n* (%)				0.27 (0.08, 0.47)	0.005
Often feels lonely	59 (14.01%)	24 (9.92%)	35 (19.55%)		
Rarely feels lonely	362 (85.99%)	218 (90.08%)	144 (80.45%)		
Declined IC, *n* (%)				0.64 (0.44, 0.84)	< 0.001
Yes	282 (66.98%)	133 (54.96%)	149 (83.24%)		
No	139 (33.02%)	109 (45.04%)	30 (16.76%)		
Intrinsic capacity items					
Vitality, *n* (%)				0.26 (0.07, 0.46)	0.006
Impaired	21 (4.99%)	6 (2.48%)	15 (8.38%)		
Not impaired	400 (95.01%)	236 (97.52%)	164 (91.62%)		
Psychological, *n* (%)				0.46 (0.27, 0.66)	< 0.001
Impaired	130 (30.88%)	53 (21.90%)	77 (43.02%)		
Not impaired	291 (69.12%)	189 (78.10%)	102 (56.98%)		
Vision, *n* (%)				0.05 (−0.14, 0.24)	0.618
Impaired	80 (19.00%)	44 (18.18%)	36 (20.11%)		
Not impaired	341 (81.00%)	198 (81.82%)	143 (79.89%)		
Hearing, *n* (%)				0.01 (−0.18, 0.20)	0.926
Impaired	95 (22.57%)	55 (22.73%)	40 (22.35%)		
Not impaired	326 (77.43%)	187 (77.27%)	139 (77.65%)		
Locomotion, *n* (%)				0.64 (0.44, 0.84)	< 0.001
Impaired	143 (33.97%)	52 (21.49%)	91 (50.84%)		
Not impaired	278 (66.03%)	190 (78.51%)	88 (49.16%)		
Cognitive function, *n* (%)				0.37 (0.18, 0.57)	< 0.001
Impaired	25 (5.94%)	5 (2.07%)	20 (11.17%)		
Not impaired	396 (94.06%)	237 (97.93%)	159 (88.83%)		
Polypharmacy, *n* (%)				0.28 (0.09, 0.48)	0.004
No	250 (59.38%)	158 (65.29%)	92 (51.40%)		
Yes	171 (40.62%)	84 (34.71%)	87 (48.60%)		
Charlson Comorbidity Index (≥3), *n* (%)				0.31 (0.11, 0.50)	0.002
No	160 (38.00%)	107 (44.21%)	53 (29.61%)		
Yes	261 (62.00%)	135 (55.79%)	126 (70.39%)		

Data are presented as mean ± SD or *n* (%). Between-group differences were assessed using the independent-samples *t*-test for continuous variables and the chi-square test or Fisher's exact test for categorical variables. Fisher's exact test was used when the expected cell count was < 10.

Table 1 presents participant characteristics stratified by tooth-count group. Compared with participants with ≥20 natural teeth, those with < 20 teeth were older (74.07 ± 6.7 vs. 69.62 ± 5.21 years, *P* < 0.001), had lower educational attainment (*P* = 0.009), lower BMI (23.66 ± 3.6 vs. 25.11 ± 3.39 kg/m^2^, *P* < 0.001), higher comorbidity burden (CCI ≥3: 70.39% vs. 55.79%, *P* = 0.002), and were more likely to experience loneliness (19.55 vs. 9.92%, *P* = 0.005). Participants with < 20 teeth also had significantly lower social support scores (53.37 ± 11.01 vs. 62.32 ± 9.11, *P* < 0.001).

## Univariate analysis

Table 2 presents results from univariate logistic regression analyses. Factors significantly associated with increased odds of IC decline included older age (OR = 1.09 per year, 95% CI: 1.05–1.13, *P* < 0.001), lower educational attainment (middle school: OR = 0.50, 95% CI: 0.28–0.90, *P* = 0.021; high school or higher: OR = 0.36, 95% CI: 0.19–0.68, *P* = 0.001), lower BMI (OR = 0.91 per unit, 95% CI: 0.85–0.96, *P* = 0.001), rarely feeling lonely (OR = 0.32, 95% CI: 0.15–0.67, *P* = 0.002), polypharmacy (OR = 2.11, 95% CI: 1.30–3.42, *P* = 0.002), higher comorbidity burden (OR = 2.16, 95% CI: 1.43–3.28, *P* < 0.001), number of natural teeth (OR = 0.93 per tooth, 95% CI: 0.91–0.95, *P* < 0.001), and lower social support scores (OR = 0.94 per point, 95% CI: 0.91–0.96, *P* < 0.0001).

**Table 2:** Univariate analysis.

Variables	Statistics	Declined IC
Teeth group, *n* (%)		
≥20	242 (57.48%)	Reference
< 20	179 (42.52%)	4.07 (2.55, 6.49), *P* < 0.0001
Gender, *n* (%)		
Male	255 (60.57%)	Reference
Female	166 (39.43%)	1.08 (0.72, 1.65), *P* = 0.7015
Age, mean ± SD	71.52 ± 6.30	1.09 (1.05, 1.13), *P* < 0.0001
Education, *n* (%)		
Primary school or lower	89 (21.14%)	Reference
Middle school	208 (49.41%)	0.50 (0.28, 0.90), *P* = 0.0216
High school or higher	124 (29.45%)	0.36 (0.19, 0.68), *P* = 0.0016
Married status, *n* (%)		
Married	372 (88.36%)	Reference
Never married	12 (2.85%)	0.36 (0.11, 1.16), *P* = 0.0876
Other (widowed/divorced/separated)	37 (8.79%)	2.17 (0.93, 5.08), *P* = 0.0743
BMI, mean ± SD	24.50 ± 3.58	0.91 (0.85, 0.96), *P* = 0.0011
Department, *n* (%)		
Gastroenterology	121 (28.74%)	Reference
Cardiology	182 (43.23%)	0.94 (0.58, 1.53), *P* = 0.8186
Endocrinology	37 (8.79%)	1.07 (0.49, 2.34), *P* = 0.8700
Urology	26 (6.18%)	1.71 (0.64, 4.58), *P* = 0.2876
Vascular surgery	32 (7.60%)	1.31 (0.56, 3.09), *P* = 0.5375
Geriatrics	23 (5.46%)	1.17 (0.45, 3.07), *P* = 0.7479
Charlson Comorbidity Index (≥3), *n* (%)		
No	160 (38.00%)	Reference
Yes	261 (62.00%)	2.16 (1.43, 3.28), *P* = 0.0003
Polypharmacy, *n* (%)		
No	250 (59.38%)	Reference
Yes	171 (40.62%)	2.11 (1.30, 3.42), *P* = 0.0024
Feelings of loneliness, *n* (%)		
Often feels lonely	59 (14.01%)	Reference
Rarely feels lonely	362 (85.99%)	0.32 (0.15, 0.67), *P* = 0.0027
Smoking status, *n* (%)		
Never smoked	234 (55.58%)	Reference
Former smoker	69 (16.39%)	0.72 (0.41, 1.26), *P* = 0.2461
Current smoker	118 (28.03%)	0.97 (0.61, 1.56), *P* = 0.9123
Drinking status, *n* (%)		
Never drinks	266 (63.18%)	Reference
Occasional drinker	61 (14.49%)	0.79 (0.44, 1.42), *P* = 0.4288
Regular drinker	94 (22.33%)	0.75 (0.46, 1.23), *P* = 0.2550
Number of natural teeth, mean ± SD	19.29 ± 9.95	0.93 (0.91, 0.95), *P* < 0.0001
Social support score, mean ± SD	58.52 ± 10.89	0.94 (0.91, 0.96), *P* < 0.0001

## Multivariable logistic regression

Table 3 presents results from hierarchical logistic regression models. In the unadjusted model, participants with fewer than 20 teeth had 4.07 times higher odds of IC decline than those with ≥20 teeth (95% CI: 2.55–6.49, *P* < 0.0001). After adjustment for sociodemographic factors (Model 2), the association remained significant but was attenuated (OR = 3.04, 95% CI: 1.82–5.07, *P* < 0.0001). In the fully adjusted model (Model 3), which additionally controlled for clinical factors, behavioral factors, and Charlson Comorbidity Index, the association remained significant (adjusted OR = 3.11, 95% CI: 1.82–5.32, *P* < 0.001).

**Table 3:** Multivariable regression analyses of the association between number of natural teeth and declined IC.

Exposure	Unadjusted	Adjusted model 2	Adjusted model 3
Number of natural teeth category	OR (95% CI), *P*-value	OR (95% CI), *P*-value	OR (95% CI), *P-*value
≥20	Reference	Reference	Reference
< 20	4.07 (2.55, 6.49), *P* < 0.0001	3.04 (1.82, 5.07), *P* < 0.0001	3.11 (1.82, 5.32), *P* < 0.001
Number of natural teeth	0.93 (0.91, 0.95), *P* < 0.0001	0.95 (0.92, 0.97), *P* < 0.0001	0.96 (0.93, 0.98), *P* = 0.001

CI, confidence interval; IC, intrinsic capacity; OR, odds ratio.

Outcome: declined intrinsic capacity. The reference category for the categorical exposure was at least 20 natural teeth.

Unadjusted model: no covariate adjustment.

Adjusted model I: adjusted for department, sex, age, education, and marital status.

Adjusted model II: adjusted for department, sex, age, education, marital status, living arrangement, smoking status, drinking status, polypharmacy (defined as at least five medications), and Charlson Comorbidity Index.

Categorical covariates were entered using indicator variables. *N* = 421; declined-IC events = 282. All *P-*values were two-sided.

**Figure 1:**
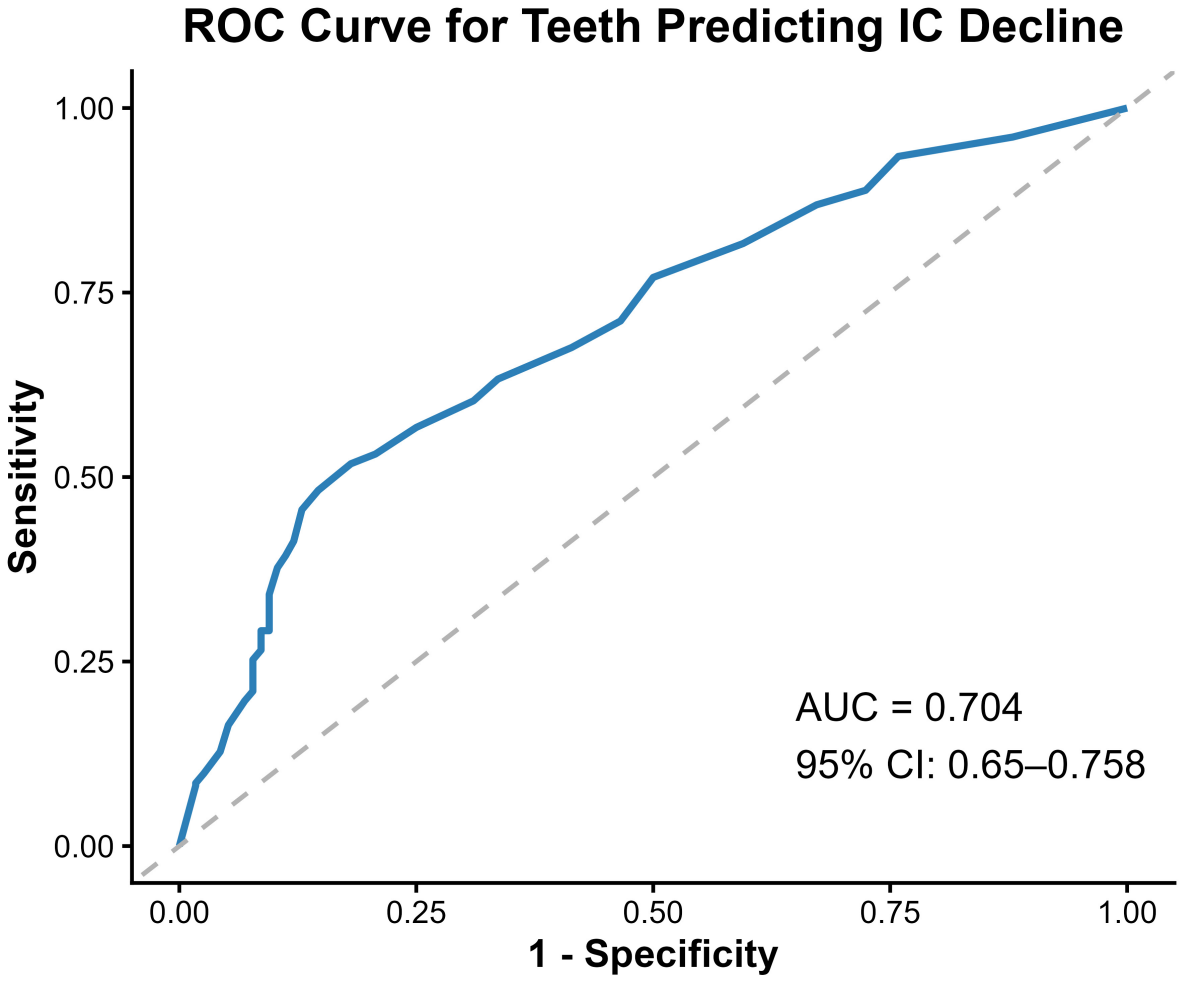
Receiver operating characteristic curve for the cross-sectional discrimination of declined intrinsic capacity by the number of natural teeth.

When the number of natural teeth was analyzed as a continuous variable, each additional tooth was associated with a 4% reduction in the odds of IC decline in the fully adjusted model (OR = 0.96 per tooth, 95% CI: 0.93–0.98, *P* = 0.001). Exploratory ROC analysis showed moderate cross-sectional discrimination for declined IC when fewer teeth were treated as the risk direction (AUC = 0.704, 95% CI: 0.605–0.758; Figure 1). This result should not be interpreted as establishing a clinically validated screening threshold.

## Sensitivity analyses

Several sensitivity analyses were performed to evaluate the robustness of the main findings. First, we applied a more stringent definition of declined intrinsic capacity (IC), defined as impairment in at least two components (IC score ≤ 4). Under this definition, the association between tooth loss and declined IC was attenuated but remained statistically significant (< 20 vs. ≥20 natural teeth: OR = 1.63, 95% CI: 1.01–2.65, *P* = 0.046; per additional tooth: OR = 0.98, 95% CI: 0.95–1.00, *P* = 0.050). Likewise, when the IC score was analyzed as a continuous outcome, the association with the number of natural teeth remained positive (β = 0.022, *P* < 0.001; Supplementary Table S1).

Second, we reconstructed IC according to the original WHO five-domain framework by combining hearing and vision into a single sensory domain. Under this alternative definition, participants with fewer than 20 natural teeth had significantly higher odds of having at least one impaired IC domain than those with ≥20 natural teeth (OR = 3.11, 95% CI: 1.82–5.33, *P* < 0.001). Each additional tooth was associated with lower odds of any IC impairment (OR = 0.95, 95% CI: 0.92–0.97, *P* < 0.001). Furthermore, associations were attenuated but remained statistically significant when declined IC was defined as impairment in at least two domains (< 20 vs. ≥20 teeth: OR = 1.97, 95% CI: 1.19–3.27, *P* = 0.009; per additional tooth: OR = 0.97, 95% CI: 0.94–0.99, *P* = 0.010). In continuous analyses, participants with fewer than 20 natural teeth had lower five-domain IC scores (β = −0.479, 95% robust CI: −0.696 to −0.263, *P* < 0.001) and a greater number of impaired domains (β = 0.479, 95% robust CI: 0.263–0.5696, *P* < 0.001). Conversely, each additional tooth was associated with higher IC scores (β = 0.023, 95% robust CI: 0.012–0.034, *P* < 0.001) and fewer impaired domains (β = −0.023, 95% robust CI: −0.034 to −0.012, *P* < 0.001; Supplementary Table S2).

Third, domain-specific analyses showed that having fewer than 20 natural teeth was significantly associated with impaired locomotion (OR = 2.28, 95% CI: 1.37–3.80, *P* = 0.008; FDR-adjusted *q* = 0.008). Each additional tooth was associated with lower odds of locomotion impairment (OR = 0.96, 95% CI: 0.93–0.98, *P* < 0.001; *q* = 0.0204) and cognitive impairment (OR = 0.93, 95% CI: 0.88–0.98, *P* = 0.005; *q* = 0.008). The association between having fewer than 20 teeth and cognitive impairment was also significant (OR = 3.81, 95% CI: 1.22–11.93, *P* = 0.022; *q* = 0.036). Associations were similar for psychological impairment. However, no significant associations were observed for vitality or sensory impairment after FDR correction (Supplementary Table S3).

Fourth, we conducted an additional sensitivity analysis using the Mini Nutritional Assessment–Short Form (MNA-SF) to define the vitality domain. Participants with fewer than 20 natural teeth had significantly lower alternative IC scores (β = −0.701, 95% robust CI: −0.980 to −0.421, *P* < 0.001) and a greater number of impaired IC components (β = 0.701, 95% robust CI: 0.421–0.980, *P* < 0.001). Similarly, each additional tooth was associated with higher IC scores (β = 0.033, 95% robust CI: 0.019–0.047, *P* < 0.001) and fewer impaired IC components (β = −0.033, 95% robust CI: −0.047 to −0.019, *P* < 0.001; Supplementary Table S4).

Overall, these sensitivity analyses indicated that the main findings were generally robust across alternative definitions of intrinsic capacity and different analytical approaches.

## Mediation analysis

**Figure 2:**
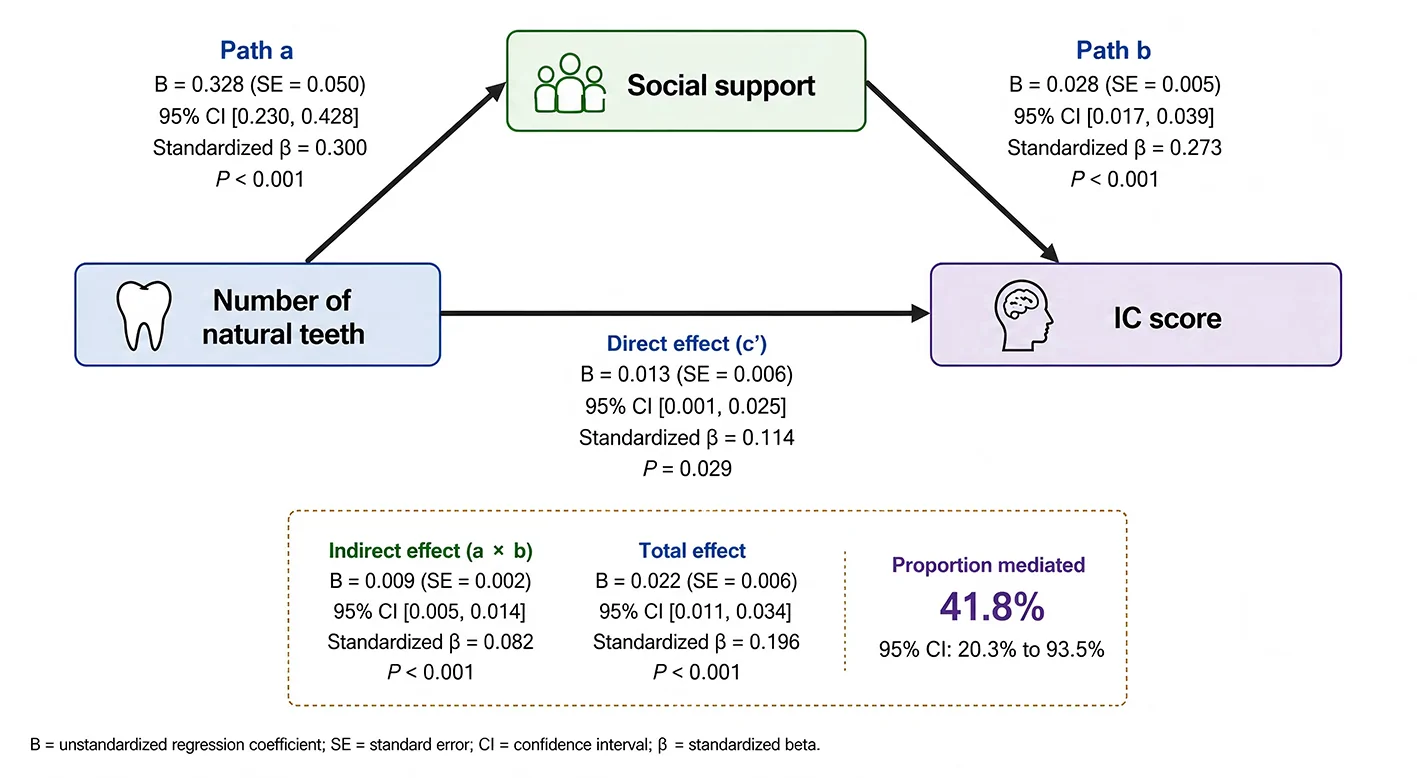
Adjusted mediation model of the association between the number of natural teeth and intrinsic capacity through social support.

A covariate-adjusted SEM was fitted to examine the exploratory indirect pathway through social support (Table 4 and Figure 2). The model used the continuous IC score from 0 to 6 as the observed outcome. Both the mediator and outcome equations were adjusted for department, sex, age, education, marital status, living arrangement, smoking status, drinking status, polypharmacy, and Charlson Comorbidity Index.

**Table 4:** Adjusted structural equation model of the indirect association between number of natural teeth and the continuous intrinsic-capacity score through social support.

Effect/path	*B*	SE	95% bootstrap CI	Standardized beta	*P*-value
Path *a*: Number of natural teeth → social support	0.328	0.050	0.230 to 0.428	0.300	< 0.001
Path *b*: social support → IC score	0.028	0.005	0.017 to 0.039	0.273	< 0.001
Direct effect (c'): number of natural teeth → IC score	0.013	0.006	0.001 to 0.025	0.114	0.029
Indirect effect (*a* × *b*)	0.009	0.002	0.005 to 0.014	0.082	< 0.001
Total effect	0.022	0.006	0.011 to 0.034	0.196	< 0.001
Descriptive indirect-to-total ratio	41.8%	NA	20.3 to 93.5%	NA	NA

*B*, unstandardized regression coefficient; CI, confidence interval; IC, intrinsic capacity; SE, standard error; SEM, structural equation modeling.

A greater number of natural teeth was associated with higher social support after covariate adjustment (path a: *B* = 0.328, SE = 0.050, 95% bootstrap CI: 0.230–0.428; standardized beta = 0.300; *P* < 0.001). Social support was positively associated with the IC score after adjustment for tooth count and covariates (path *b*: *B* = 0.028, SE = 0.005, 95% bootstrap CI: 0.017–0.039; standardized beta = 0.273; *P* < 0.001).

The adjusted direct association between the number of natural teeth and the IC score was *B* = 0.013 (SE = 0.006, 95% bootstrap CI: 0.001–0.025; standardized beta = 0.114; *P* = 0.029). The indirect association through social support was *B* = 0.009 (SE = 0.002, 95% bootstrap CI: 0.005–0.014; standardized beta = 0.082; *P* < 0.001), and the total association was *B* = 0.022 (SE = 0.006, 95% bootstrap CI: 0.011–0.034; standardized beta = 0.196; *P* < 0.001).

The descriptive indirect-to-total ratio was 41.8% (95% bootstrap interval: 20.3%−93.5%) and was not interpreted as a causal proportion mediated. The model explained 27.2% of the variance in social support and 22.4% of the variance in the IC score. The model was saturated (scaled chi-square = 0.00, df = 0; CFI = 1.000; TLI = 1.000; SRMR approximately 0.000; RMSEA not estimable; AIC = 4,314.79; BIC = 4,456.29); therefore, the global fit indices were not diagnostically informative.

The IC outcome was the observed continuous score ranging from 0 to 6, with higher scores indicating better intrinsic capacity.

The binary declined-IC variable was not modeled in the SEM. Logistic regression was used for the clinically defined binary threshold, whereas the exploratory SEM retained the full 0–6 score to avoid information loss and to estimate the indirect association as a product of linear coefficients on a common scale.

The model adjusted both the mediator and outcome equations for hospital service, sex, age, education, marital status, living arrangement, smoking status, drinking status, polypharmacy (at least five medications), and Charlson Comorbidity Index.

Point estimates, robust SEs, standardized coefficients, and *P*-values were obtained using robust maximum likelihood estimation (MLR). Confidence intervals were estimated from 5,000 non-parametric bootstrap resamples.

Model information: *N* = 421; scaled chi-square = 0.00, df = 0; CFI = 1.000; TLI = 1.000; SRMR = 0.000; RMSEA not estimable; AIC = 4,314.80; BIC = 4,456.29. R-squared = 0.272 for social support and R-squared = 0.224 for IC score.

Because this observed-variable path model was saturated (df = 0), conventional global fit indices are expected to indicate perfect fit and should not be interpreted as independent evidence of model adequacy.

The descriptive indirect-to-total ratio is presented descriptively with a bootstrap percentile interval. It is not interpreted as a causal proportion mediated because exposure, mediator, and outcome were measured cross-sectionally.

## Discussion

In this cross-sectional study of 421 hospitalized older adults in China, fewer natural teeth were associated with declined intrinsic capacity after adjustment for sociodemographic, clinical, behavioral, and chronic disease factors. Participants with fewer than 20 natural teeth had approximately three times the odds of having at least one impaired IC component compared with those with ≥20 natural teeth. In exploratory SEM, social support statistically accounted for part of the association between number of teeth and IC score. These findings should be interpreted as cross-sectional associations and indirect pathways, not as evidence that tooth loss causes IC decline or that social support lies on a confirmed temporal causal pathway.

Our findings align with and extend a growing body of literature examining the relationship between oral health and intrinsic capacity in older adults. Recent studies using NHANES data have documented that tooth loss (14) and periodontal disease (15) are associated with declined IC across multiple domains, particularly locomotion and nutritional capacity, among U.S. older populations. Similarly, research from Japanese frailty clinics has shown that oral frailty independently predicts comprehensive IC impairment (27). These cross-cultural findings mirror our observation that fewer natural teeth are associated with more than twice the odds of IC decline among hospitalized Chinese older adults (adjusted OR = 3.11, 95% CI: 1.82–5.32).

However, our study extends previous research in two important ways. First, while prior investigations have focused primarily on establishing associations, we used SEM to examine whether social support statistically accounted for part of the dental status-IC relationship. The adjusted indirect pathway suggests that psychosocial resources may be relevant to the co-occurrence of fewer teeth and lower IC, alongside biological and nutritional mechanisms such as inflammation and malnutrition. This interpretation remains exploratory because temporal order could not be established. Second, our study population, hospitalized older adults in China, represents a high-risk group often underrepresented in population-based surveys. Acute illness, multimorbidity, medication burden, nutritional vulnerability, and reduced opportunities for social participation may make the oral health-IC association especially clinically relevant in inpatient settings. Furthermore, the findings were generally robust under stricter binary thresholds, continuous IC-score analyses, the WHO-aligned five-domain IC structure, and the MNA-SF-based vitality definition. These results suggest that the association between tooth loss and IC decline was not driven solely by a single operational definition of IC.

The observed indirect pathway through social support is consistent with emerging evidence on the psychosocial consequences of tooth loss (28). Qualitative studies have reported that older adults with missing teeth may experience embarrassment, reduced self-confidence, and avoidance of social situations involving eating or speaking (29, 30). These psychosocial impacts may be linked to smaller social networks and lower perceived support, which in turn may relate to physical and mental health. Previous research has similarly linked low social support to incident IC decline (31). Recent work has also emphasized oral health as a multidimensional determinant of healthy aging and frailty, with potential biological, functional, and psychosocial pathways, including social isolation (32). Together, these findings support considering oral health and social resources within multidimensional aging frameworks, while avoiding causal claims from cross-sectional data.

## Clinical and public health implications

Our findings have several important implications for clinical practice and public health policy. Because dental status was associated with IC in this inpatient sample, oral health information may be useful within comprehensive geriatric assessment (33). Simple measures such as counting natural teeth or asking about masticatory difficulty can help characterize oral health burden, but the exploratory AUC of 0.704 indicates only moderate cross-sectional discrimination and does not establish clinical screening utility. For older adults with significant tooth loss, dental rehabilitation (e.g., dentures or implants) may improve masticatory function and psychosocial wellbeing. However, prospective and interventional studies are needed to determine whether such approaches contribute to the preservation of intrinsic capacity. Similarly, the association involving social support suggests a potential psychosocial intervention target, but the present data cannot show that enhancing social support prevents IC decline (24). Healthcare providers may consider assessing social support among patients with tooth loss and connecting those with inadequate support to appropriate resources. Public health campaigns should emphasize preserving natural teeth throughout the lifespan as one component of broader healthy aging strategies (34).

## Strengths and limitations

This study has several strengths. First, we employed the comprehensive IC framework recommended by WHO, providing a multidimensional assessment of functional capacity. Second, we used validated instruments for all key measurements, supporting reliability and comparability with other studies. Third, we considered a comprehensive set of potential confounders, including comorbidity burden and polypharmacy. Fourth, we conducted extensive sensitivity analyses to evaluate the robustness of the findings. The association was examined using categorical and continuous measures of tooth count; binary, continuous, and WHO-aligned five-domain IC outcomes; an alternative MNA-SF-based vitality definition; and models excluding vitality to address potential mathematical coupling with BMI. Results were also evaluated across alternative covariate sets and individual IC domains, with correction for multiple testing. The broadly consistent associations across these specifications, particularly for any IC impairment and the locomotion domain, reduce the likelihood that the primary finding was driven solely by a particular IC definition, exposure threshold, covariate set, or vitality indicator. Complete data for all modeled variables, transparent assessment of model complexity, and convergence of all regression models further strengthen the reliability of the analyses. Attenuation under stricter multiple-impairment definitions suggests that the association may vary according to the severity and composition of IC decline, although these robustness analyses do not overcome the causal limitations of the cross-sectional design. Finally, SEM provided an exploratory assessment of an indirect pathway through social support.

However, several limitations should be acknowledged. First, the cross-sectional design precludes causal inference and prevents establishment of the temporal order among tooth loss, social support, and IC. Reverse or bidirectional relationships are plausible because declining IC may reduce an individual's ability to maintain oral hygiene or obtain dental care. Consequently, the indirect association identified in the SEM should not be interpreted as causal mediation. Second, IC was operationalized using study-specific indicators available from the inpatient assessment rather than a complete validated WHO ICOPE instrument. Handgrip strength does not capture gait speed, balance, or broader mobility; BMI alone does not capture appetite loss, recent weight loss, or other dimensions of vitality; and vision and hearing were self-reported rather than objectively assessed.

Third, the study included hospitalized older adults from a single tertiary hospital. These participants may have had greater multimorbidity, medication burden, and functional vulnerability than community-dwelling populations. In addition, excluding individuals with severe cognitive impairment, terminal illness, or an inability to complete the assessment may have resulted in selection of a relatively less impaired inpatient sample. These factors limit generalizability to community populations, more severely impaired patients, and other geographic or cultural settings.

Fourth, residual and unmeasured confounding remains possible despite adjustment based on a pre-specified conceptual framework. Potential unmeasured factors include lifetime socioeconomic circumstances, dietary intake, oral hygiene practices, dental-care utilization, systemic inflammation, and genetic or early-life determinants. Social support and loneliness may also represent mediators, consequences, or proxies for broader social conditions, making estimates from models including these variables difficult to interpret causally. Fifth, the number of natural teeth is a limited measure of oral health and does not fully characterize functional dentition. Information was unavailable on dentures, prosthetic rehabilitation, occlusal support, periodontal disease, causes and timing of tooth loss, dental caries, and objective masticatory performance.

Sixth, several behavioral and clinical variables, including loneliness, smoking, alcohol consumption, and sensory impairments, were self-reported and may be affected by recall or reporting bias. Finally, although all regression models converged and no observations were excluded because of missing data, the ratio of non-events to model parameters in the fully adjusted logistic model was borderline, so some risk of overfitting and imprecise estimation remains (Supplementary Table S5). Prospective multicenter studies using standardized dental examinations, comprehensive IC assessments, and repeated measurements are needed to clarify temporal and potentially bidirectional relationships. Intervention studies are subsequently required to determine whether dental rehabilitation, social-support enhancement, or combined approaches can preserve IC.

## Conclusions

In this cross-sectional study, fewer natural teeth were associated with declined intrinsic capacity among hospitalized older adults in China, and social support statistically accounted for part of this association. These findings support considering oral health as one component of multidimensional healthy aging assessment and suggest that psychosocial resources may be relevant when evaluating older adults with tooth loss. Longitudinal and interventional studies are needed to determine whether improving oral health, enhancing social support, or combining both approaches can help preserve intrinsic capacity.

## Data Availability

The raw data supporting the conclusions of this article will be made available by the authors, without undue reservation.
