# Supplementary material for: Number of natural teeth and intrinsic capacity among hospitalized older adults in China: a cross-sectional study on the role of social support
Source: Front Med (Lausanne). 2026 Aug 21;13:1848888. doi: 10.3389/fmed.2026.1848888 (PMC13542646; doi:10.3389/fmed.2026.1848888)
Supplement: Supplementary file 1 [file Table_1.docx]

**Supplementary Table S1. Sensitivity analyses using an alternative intrinsic capacity cut-point and the continuous intrinsic capacity score**

| **Sensitivity outcome** | **Exposure** | **Measure** | **Estimate (95% CI)** | **P value** | **N** | **Events** |
| --- | --- | --- | --- | --- | --- | --- |
| At least 2 impaired components (IC score <=4) | <20 vs. >=20 natural teeth | OR | 1.63 (1.01 to 2.65) | 0.046 | 421 | 141 |
| At least 2 impaired components (IC score <=4) | Per additional natural tooth | OR | 0.98 (0.95 to 1.00) | 0.050 | 421 | 141 |
| Continuous IC score (0-6; higher score indicates better IC) | <20 vs. >=20 natural teeth | B | -0.459 (-0.687 to -0.231) | <0.001 | 421 | NA |
| Continuous IC score (0-6; higher score indicates better IC) | Per additional natural tooth | B | 0.022 (0.010 to 0.034) | <0.001 | 421 | NA |

Values are fully adjusted estimates. The final adjustment set was department, sex, age, education, marital status, living arrangement, smoking status, drinking status, polypharmacy (defined as use of at least five medications), and Charlson Comorbidity Index.

BMI, social support, and loneliness were not included in these fully adjusted sensitivity models.

Abbreviations: B, unstandardized coefficient; CI, confidence interval; IC, intrinsic capacity; MNA-SF, Mini Nutritional Assessment Short Form; OR, odds ratio.

**Supplementary Table S2. Sensitivity analyses using a five-domain intrinsic capacity definition**

| **Sensitivity outcome** | **Exposure** | **Measure** | **Estimate (95% CI)** | **P value** | **N** | **Events** |
| --- | --- | --- | --- | --- | --- | --- |
| Any impaired WHO domain (five-domain score <=4) | <20 vs. >=20 natural teeth | OR | 3.11 (1.82 to 5.33) | <0.001 | 421 | 282 |
| At least 2 impaired WHO domains (five-domain score <=3) | <20 vs. >=20 natural teeth | OR | 1.97 (1.19 to 3.27) | 0.009 | 421 | 125 |
| Five-domain IC score (0-5; higher is better) | <20 vs. >=20 natural teeth | B | -0.479 (-0.696 to -0.263) | <0.001 | 421 | NA |
| Count of impaired WHO domains (0-5; higher is worse) | <20 vs. >=20 natural teeth | B | 0.479 (0.263 to 0.696) | <0.001 | 421 | NA |
| Any impaired WHO domain (five-domain score <=4) | Per additional natural tooth | OR | 0.95 (0.92 to 0.97) | <0.001 | 421 | 282 |
| At least 2 impaired WHO domains (five-domain score <=3) | Per additional natural tooth | OR | 0.97 (0.94 to 0.99) | 0.010 | 421 | 125 |
| Five-domain IC score (0-5; higher is better) | Per additional natural tooth | B | 0.023 (0.012 to 0.034) | <0.001 | 421 | NA |
| Count of impaired WHO domains (0-5; higher is worse) | Per additional natural tooth | B | -0.023 (-0.034 to -0.012) | <0.001 | 421 | NA |

Values are fully adjusted estimates. The final adjustment set was department, sex, age, education, marital status, living arrangement, smoking status, drinking status, polypharmacy (defined as use of at least five medications), and Charlson Comorbidity Index.

BMI, social support, and loneliness were not included in these fully adjusted sensitivity models.

Abbreviations: B, unstandardized coefficient; CI, confidence interval; IC, intrinsic capacity; MNA-SF, Mini Nutritional Assessment Short Form; OR, odds ratio.

**Supplementary Table S3. Adjusted associations between number of natural teeth and impairment in each WHO intrinsic-capacity domain**

| **WHO IC domain outcome** | **Exposure** | **OR (95% CI)** | **P value** | **FDR q value** | **N** | **Events** |
| --- | --- | --- | --- | --- | --- | --- |
| Locomotion impairment | <20 vs. >=20 natural teeth | 2.28 (1.37 to 3.80) | 0.002 | 0.008 | 421 | 143 |
| Locomotion impairment | Per additional natural tooth | 0.96 (0.93 to 0.98) | <0.001 | 0.004 | 421 | 143 |
| Cognitive impairment | <20 vs. >=20 natural teeth | 3.81 (1.22 to 11.93) | 0.022 | 0.036 | 421 | 25 |
| Cognitive impairment | Per additional natural tooth | 0.93 (0.88 to 0.98) | 0.005 | 0.008 | 421 | 25 |
| Vitality impairment | <20 vs. >=20 natural teeth | 3.09 (0.98 to 9.73) | 0.054 | 0.067 | 421 | 21 |
| Vitality impairment | Per additional natural tooth | 0.96 (0.91 to 1.01) | 0.139 | 0.174 | 421 | 21 |
| Psychological impairment | <20 vs. >=20 natural teeth | 2.02 (1.22 to 3.34) | 0.006 | 0.015 | 421 | 130 |
| Psychological impairment | Per additional natural tooth | 0.96 (0.94 to 0.99) | 0.004 | 0.008 | 421 | 130 |
| Sensory impairment (vision or hearing) | <20 vs. >=20 natural teeth | 1.32 (0.83 to 2.10) | 0.249 | 0.249 | 421 | 148 |
| Sensory impairment (vision or hearing) | Per additional natural tooth | 1.00 (0.97 to 1.02) | 0.674 | 0.674 | 421 | 148 |

Values are fully adjusted estimates. The final adjustment set was department, sex, age, education, marital status, living arrangement, smoking status, drinking status, polypharmacy (defined as use of at least five medications), and Charlson Comorbidity Index.

BMI, social support, and loneliness were not included in these fully adjusted sensitivity models.

Abbreviations: B, unstandardized coefficient; CI, confidence interval; IC, intrinsic capacity; MNA-SF, Mini Nutritional Assessment Short Form; OR, odds ratio.

FDR q values were calculated across the five domain-specific outcomes separately for each exposure parameterization.

**Supplementary Table S4. Sensitivity analyses replacing BMI-defined vitality with MNA-SF-defined vitality**

| **IC definition** | **Sensitivity outcome** | **Exposure** | **Measure** | **Estimate (95% CI)** | **P value** | **N** | **Events** |
| --- | --- | --- | --- | --- | --- | --- | --- |
| Six-component IC with MNA-SF vitality | Any impaired component (score <=5) | <20 vs. >=20 natural teeth | OR | 4.06 (2.19 to 7.50) | <0.001 | 421 | 305 |
| Six-component IC with MNA-SF vitality | At least 2 impaired components (score <=4) | <20 vs. >=20 natural teeth | OR | 2.75 (1.72 to 4.39) | <0.001 | 421 | 205 |
| Six-component IC with MNA-SF vitality | Alternative IC score (0-6; higher is better) | <20 vs. >=20 natural teeth | B | -0.701 (-0.980 to -0.421) | <0.001 | 421 | NA |
| Six-component IC with MNA-SF vitality | Count of impaired components (0-6; higher is worse) | <20 vs. >=20 natural teeth | B | 0.701 (0.421 to 0.980) | <0.001 | 421 | NA |
| Six-component IC with MNA-SF vitality | Any impaired component (score <=5) | Per additional natural tooth | OR | 0.93 (0.90 to 0.96) | <0.001 | 421 | 305 |
| Six-component IC with MNA-SF vitality | At least 2 impaired components (score <=4) | Per additional natural tooth | OR | 0.96 (0.94 to 0.98) | <0.001 | 421 | 205 |
| Six-component IC with MNA-SF vitality | Alternative IC score (0-6; higher is better) | Per additional natural tooth | B | 0.033 (0.019 to 0.047) | <0.001 | 421 | NA |
| Six-component IC with MNA-SF vitality | Count of impaired components (0-6; higher is worse) | Per additional natural tooth | B | -0.033 (-0.047 to -0.019) | <0.001 | 421 | NA |
| Five-domain IC with MNA-SF vitality | Any impaired WHO domain (score <=4) | <20 vs. >=20 natural teeth | OR | 4.06 (2.19 to 7.50) | <0.001 | 421 | 305 |
| Five-domain IC with MNA-SF vitality | At least 2 impaired WHO domains (score <=3) | <20 vs. >=20 natural teeth | OR | 3.31 (2.05 to 5.33) | <0.001 | 421 | 191 |
| Five-domain IC with MNA-SF vitality | Alternative five-domain IC score (0-5; higher is better) | <20 vs. >=20 natural teeth | B | -0.721 (-0.990 to -0.452) | <0.001 | 421 | NA |
| Five-domain IC with MNA-SF vitality | Count of impaired WHO domains (0-5; higher is worse) | <20 vs. >=20 natural teeth | B | 0.721 (0.452 to 0.990) | <0.001 | 421 | NA |
| Five-domain IC with MNA-SF vitality | Any impaired WHO domain (score <=4) | Per additional natural tooth | OR | 0.93 (0.90 to 0.96) | <0.001 | 421 | 305 |
| Five-domain IC with MNA-SF vitality | At least 2 impaired WHO domains (score <=3) | Per additional natural tooth | OR | 0.95 (0.92 to 0.97) | <0.001 | 421 | 191 |
| Five-domain IC with MNA-SF vitality | Alternative five-domain IC score (0-5; higher is better) | Per additional natural tooth | B | 0.035 (0.021 to 0.048) | <0.001 | 421 | NA |
| Five-domain IC with MNA-SF vitality | Count of impaired WHO domains (0-5; higher is worse) | Per additional natural tooth | B | -0.035 (-0.048 to -0.021) | <0.001 | 421 | NA |

Values are fully adjusted estimates. The final adjustment set was department, sex, age, education, marital status, living arrangement, smoking status, drinking status, polypharmacy (defined as use of at least five medications), and Charlson Comorbidity Index.

BMI, social support, and loneliness were not included in these fully adjusted sensitivity models.

Abbreviations: B, unstandardized coefficient; CI, confidence interval; IC, intrinsic capacity; MNA-SF, Mini Nutritional Assessment Short Form; OR, odds ratio.

MNA-SF 12-14 was defined as preserved vitality; MNA-SF 0-11 was defined as impaired vitality or malnutrition risk.
